# Entacapone Treatment Modulates Hippocampal Proteins Related to Synaptic Vehicle Trafficking

**DOI:** 10.3390/cells9122712

**Published:** 2020-12-18

**Authors:** Dae Young Yoo, Hyo Young Jung, Woosuk Kim, Kyu Ri Hahn, Hyun Jung Kwon, Sung Min Nam, Jin Young Chung, Yeo Sung Yoon, Dae Won Kim, In Koo Hwang

**Affiliations:** 1 Department of Anatomy and Cell Biology, Research Institute for Veterinary Science, College of Veterinary Medicine, Seoul National University, Seoul 08826, Korea; dyyoo@sch.ac.kr (D.Y.Y.); hyoyoung@snu.ac.kr (H.Y.J.); tank3430@hallym.ac.kr (W.K.); hkinging@snu.ac.kr (K.R.H.); ysyoon@snu.ac.kr (Y.S.Y.); 2 Department of Anatomy, College of Medicine, Soonchunhyang University, Cheonan 31151, Korea; 3 Department of Biomedical Sciences, Research Institute for Bioscience and Biotechnology, Hallym University, Chuncheon 24252, Korea; 4 Department of Biochemistry and Molecular Biology, Research Institute of Oral Sciences, College of Dentistry, Gangneung-Wonju National University, Gangneung 25457, Korea; donuts25@gwnu.ac.kr; 5 Department of Anatomy, School of Medicine and Institute for Environmental Science, Wonkwang University, Iksan 54538, Korea; namvet1@wku.ac.kr; 6 Department of Veterinary Internal Medicine and Geriatrics, College of Veterinary Medicine, Kangwon National University, Chuncheon 24341, Korea; jychung77@gmail.com

**Keywords:** entacapone, hippocampus, proteomics, synaptic trafficking, mouse

## Abstract

Entacapone, a reversible inhibitor of catechol-O-methyl transferase, is used for patients in Parkinson’s disease because it increases the bioavailability and effectiveness of levodopa. In the present study, we observed that entacapone increases novel object recognition and neuroblasts in the hippocampus. In the present study, two-dimensional electrophoresis (2-DE) and matrix-assisted laser desorption/ionization time-of-flight (MALDI-TOF) mass spectrometry were performed to compare the abundance profiles of proteins expressed in the hippocampus after entacapone treatment in mice. Results of 2-DE, MALDI-TOF mass spectrometry, and subsequent proteomic analysis revealed an altered protein expression profile in the hippocampus after entacapone treatment. Based on proteomic analysis, 556 spots were paired during the image analysis of 2-DE gels and 76 proteins were significantly changed more than two-fold among identified proteins. Proteomic analysis indicated that treatment with entacapone induced expressional changes in proteins involved in synaptic transmission, cellular processes, cellular signaling, the regulation of cytoskeletal structure, energy metabolism, and various subcellular enzymatic reactions. In particular, entacapone significantly increased proteins related to synaptic trafficking and plasticity, such as dynamin 1, synapsin I, and Munc18-1. Immunohistochemical staining showed the localization of the proteins, and western blot confirmed the significant increases in dynamin I (203.5% of control) in the hippocampus as well as synapsin I (254.0% of control) and Munc18-1 (167.1% of control) in the synaptic vesicle fraction of hippocampus after entacapone treatment. These results suggest that entacapone can enhance hippocampal synaptic trafficking and plasticity against various neurological diseases related to hippocampal dysfunction.

## 1. Introduction

The hippocampus, one of the major components of the limbic system, plays essential roles in the integration of spatial memory [1] and aversive contextual memory [2]. In addition, the hippocampus is a neurogenic region, including the subventricular zone, and new neurons are associated with object recognition performance in the hippocampus [3]. Several studies have demonstrated that damage to the hippocampus causes impairment in object recognition performance [4,5], and novel object recognition is inhibited by blocking neurogenesis in the hippocampus [6]. The hippocampus receives synaptic inputs from various brain regions, including the entorhinal cortex, hypothalamus, amygdala, ventral tegmental area, and locus coeruleus [7,8]. Among these connections, the dopaminergic system is very important because dopamine receptors are expressed in the hippocampus and dopaminergic transmission is associated with learning, memory, and synaptic plasticity [9,10].

Entacapone [(2*E*)-2-cyano-3-(3,4-dihydroxy-5-nitrophenyl)-*N*,*N*-diethylprop-2-enamide] is known as a reversible inhibitor of catechol-*O*-methyl transferase (COMT), and entacapone increases the bioavailability and effectiveness of levodopa [11]. Several in vitro studies have shown that entacapone decreases oxidative stress [12] and can quench oxidative radicals (ONOO^−^ and HOCl^−^), and effectively ameliorates H_2_O_2_-induced cell death in human umbilical vein endothelial cells [13]. In addition, entacapone decreases aggregation of α-synuclein and β-amyloid [14]. In in vivo studies, oral administration of entacapone is promptly absorbed, reaches its maximal concentration at 0.7–1.82 h after treatment, and is eliminated by 4 h after treatment [15,16]. Treatment with 30–100 mg/kg entacapone reduces COMT activity [17], increases the formation of 3,4-dihydroxyphenylacetic acid in the striatum [18], and decreases the efflux of homovanillic acid [17], although entacapone does not show any penetration efficacy in the blood-brain barrier [19]. In a previous study, we demonstrated that entacapone treatment significantly increased novel object recognition memory and hippocampal neurogenesis by upregulating brain-derived neurotrophic factor, tyrosine kinase receptor B, and phosphorylated cAMP response element-binding protein [20].

However, to date, there are no considerable studies on the effects of entacapone on protein expression in the hippocampus. In the present study, therefore, we investigated protein expression following entacapone treatment using two-dimensional D-gel electrophoresis (2DE) followed by matrix-assisted laser desorption/ionization time-of-flight mass spectrometry (MALDI-TOF MS) in the hippocampus. In addition, we validated the significantly altered proteins related to synaptic vesicle trafficking in the hippocampus.

## 2. Materials and Methods

### 2.1. Chemicals

Alfaxalone and xylazine were obtained from Careside (Seongnam, South Korea) and Bayer Korea (Seoul, South Korea), respectively. Buffers or reagents used in proteomic and immunohistochemical study such as urea, thiourea, 3-[(3-cholamidopropyl) dimethylammonio]-1-propanesulfonate, dithioerythritol, Tris buffer, sodium dodecyl sulfate (SDS), glyceride, acrylamide, Tributylphosphine, ammonium bicarbonate, acetonitrile, ammonium bicarbonate, paraformaldehyde, phosphate buffer. Syn-PER Reagent and nitrocellulose membranes were purchased from Thermo Fisher Scientific (Waltham, MA, USA) and Pall Crop (East Hills, NY, USA), respectively, Antibodies specific for dynamin 1, Munc18-1, and synapsin I purchased from Abcam (Cambridge, UK) and normal goat serum and FITC-conjugated goat anti-rabbit IgG were acquired from Vector Lab. (Burlingame, CA, USA) and Jackson ImmunoResearch (West Grove, PA, USA), respectively.

### 2.2. Experimental Animals and Entacapone Treatment

Male seven-week-old C57BL/6J mice (*n* = 60) were obtained from Orient Bio (Sungnam, South Korea). Animals were housed in a specific pathogen-free facility at the Seoul National University College of Veterinary Medicine under adequate temperature, humidity, and light/dark cycle. Experimental protocols for animal experiments were approved by the Institutional Animal Care and Use Committee (Approval no. SNU-130730-1).

### 2.3. Entacapone Treatment

After acclimating for 1 week, mice (*n* = 30 in each group) were divided into a vehicle-treated group and a 50 mg/kg entacapone-treated group. Entacapone was dissolved in 0.9% physiological saline, and vehicle or entacapone was administered orally to the mice once a day for 21 days using a feeding needle.

### 2.4. Protein Preparation for 2DE

Two hours after the last entacapone or vehicle treatment, mice were deeply anesthetized with a mixture of 75 mg/kg alfaxalone and 10 mg/kg xylazine. Hippocampal tissues, which were isolated from the brains of vehicle- or entacapone-treated mice (*n* = 20 in each group), were processed as described in previous studies [21,22]. Suspensions were sonicated 5 times for 10 s and were centrifuged at 45,000 rpm for 45 min. Proteins in the supernatants were quantified using a 2D Quant kit (GE Healthcare, Uppsala, Sweden).

### 2.5. Analysis of 2-DE Gels

As previously described [21,22], 2-DE was performed. Briefly, from each group, 1 mg of hippocampal protein was rehydrated in the sample buffer (7 M urea, 2 M thiourea, 4.5% 3-[(3-cholamidopropyl) dimethylammonio]-1-propanesulfonate, 100 mM dithioerythritol, 40 mM Tris, pH 8.8), and applied to immobilized pH 3–10 non-linear gradient strips (Amersham Biosciences, Uppsala, Sweden). Isoelectric focusing was performed at 80,000 Vh. The strips were reduced and alkylated in Tributylphosphine equilibration buffer (6 M urea, 2% sodium dodecyl sulfate (SDS), 30 mM Tris, 20% glycerol, 2.5% acrylamide solution, and 5 mM Tributylphosphine), and then proteins were separated in the second dimension using SDS-polyacrylamide gel electrophoresis (SDS-PAGE) (9–16%) at 40 mA for 5 h. Proteins were fixed in 40% methanol and 5% phosphoric acid for 1 h, and the gels were stained with Coomassie brilliant blue G 250 solution overnight. The gels were destained with ultrapure distilled water. After fixation, the gels were scanned using a GS710 scanning densitometer (Bio-Rad, Richmond, CA, USA), and the results were converted into electronic files. The data were analyzed with the Image Master Platinum 5.0 image analysis program (Amersham Biosciences).

### 2.6. Trypsin Digestion of Master Gels

Protein spots of interest were picked from the preparative gel into 1.5-mL Eppendorf tubes. The gel pieces were washed with distilled water and shaken in 50 mM ammonium bicarbonate and acetonitrile (6:4) until Coomassie brilliant blue G 250 was washed out. Proteins in the spots (100 ng per spot) were digested on ice with 5 μL trypsin (Promega, Southhampton, UK) in 50 mM ammonium bicarbonate for 45 min, and then incubated at 37 °C for 12 h.

### 2.7. Protein Identification Using MALDI-TOF MS

MALDI-TOF MS was performed as described previously [21,22]. Tryptic peptides were desalted and purified using the Poros 10 R2 resin (Applied Biosystems, Foster City, CA, USA) and Oligo R3 (Applied Biosystems). Mass spectra of peptides were obtained using the 4800 MALDI-TOF analyzer (Applied Biosystems) in reflection/delayed extraction mode with an accelerating voltage of 20 kV, and the data were summed from 500 laser pulses. The spectrum was calibrated with trypsin auto-digested peaks (m/z: 842.5090 and 2211.1046), and monoisotopic peptide masses were obtained with the Data Explorer 4.4 (PerSeptive Biosystems Inc., Framingham, MA, USA). A mass range of 800–4000 m/z was used with 1000 shots per spectrum. At the end of the macro process, raw data were generated about the centroid mass, resolution, height, and S/N ratio of each peak. These data were converted to an Excel file and were used for MASCOT (Matrix Science, London, UK) search to identify peptide sequences that were present in the protein sequence database NCBInr (mouse). Protein scores higher than 67 were considered significant (*p* < 0.05).

### 2.8. Validation of Selected Proteins

#### 2.8.1. Western Blot Analyses

To validate the proteins related to synaptic vehicles selected from 2-DE and MALDI-TOF MS, mice (*n* = 5 per group) were euthanized with a mixture of alfaxalone and xylazine. The left and right hippocampal tissues were quickly removed from the brain and left hippocampi were homogenized in a buffer, as described previously [21,22], while the right hippocampi were treated with Syn-PER Reagent and homogenized. The homogenate was centrifuged at 15,000× *g* for 20 min to obtain the synaptosome fraction. Briefly, protein-transferred nitrocellulose membranes were sequentially treated with rabbit anti-dynamin 1 (1:500, Abcam), rabbit anti-Munc18-1 (1:1000), and rabbit anti-synapsin I (1:500). Data were normalized to β-actin levels as demonstrated in previous studies [21,22].

#### 2.8.2. Immunohistochemistry

To visualize the proteins related to synaptic vehicles selected from 2-DE and MALDI-TOF MS, mice (*n* = 5 per group) were anesthetized with a mixture of alfaxalone and xylazine and perfused transcardially with 0.9% saline and 4% paraformaldehyde in phosphate buffer. Serially sectioned tissues (30-μm thickness) were used for immunohistochemical staining for dynamin 1, synapsin I, and Munc18-1 between 1.7 and 2.3 mm caudal to the bregma, referring to the mouse atlas by Paxinos and Franklin [23]. Briefly, three sections, 150 μm apart, were selected and sequentially treated with 5% normal goat serum (Vector Lab.), primary antibodies, and FITC-conjugated goat anti-rabbit IgG (1:200; Jackson ImmunoResearch). The optical density of dynamin 1, synapsin I, and Munc18-1 was calculated using ImageJ software (National Institutes of Health, Bethesda, MD, USA) as described previously [20]. Briefly, the density of immunoreactive structures was measured based on 256 gray scale and the sum of density of pixel × pixel number was calculated in three sections per animal. The relative optical density was expressed as percentages of the vehicle-treated group.

### 2.9. Statistical Analysis

Results are shown as mean ± standard deviation. Results were statistically analyzed with Student’s *t*-test using SPSS (IBM, Armonk, NY, USA). A value of *p* < 0.05 was considered statistically significant.

## 3. Results

### 3.1. Proteomic Profiles Changed by Entacapone Treatment

**Figure 1:**
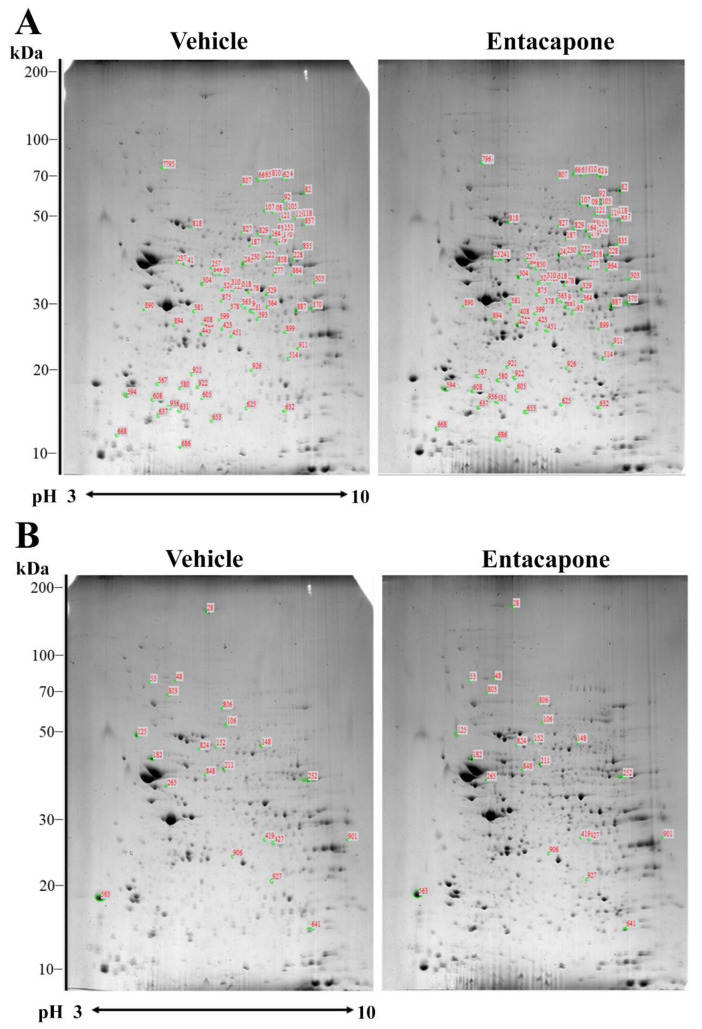
Two-dimensional electrophoresis (2-DE) gel pattern of hippocampal proteins extracted from the vehicle- and entacapone-treated groups. The spots labeled with red numbers indicate that the expression level of corresponding proteins is upregulated (**A**) or downregulated (**B**) more than two-fold following entacapone treatment. Proteins extracted from both groups were applied to immobilized pH 3–10 non-linear gradient strips, and then were separated with 9–16% sodium dodecyl sulfate (SDS)-polyacrylamide gels. Gels were stained with Coomassie brilliant blue G 250 solution.

To elucidate significantly altered protein expression caused by entacapone treatment, proteomic analysis was performed using the hippocampi of vehicle- and 50 mg/kg entacapone-treated mice. Figure 1 shows representative 2-DE gel images with separated protein spots obtained from the hippocampi of vehicle- and entacapone-treated mice. In both groups, more than 600 protein spots were detected on each 2-DE gel, and every spot was located within the ranges of pH 3–10 and 10–200 kDa. In this study, 556 spots were paired during the image analysis of 2-DE gels (Figure 1). Image analysis revealed that the expression of 106 protein spots had more than a 2.0-fold difference, and 76 proteins, except for unnamed and clustered proteins, were identified with MALDI-TOF MS and the subsequent MASCOT search. Identified proteins were classified according to the following criteria: (1) proteins involved in vesicular trafficking and endocytosis (Table 1); (2) proteins involved in cellular signaling and various cellular processes including cell cycle, mitosis, and differentiation (Table 2); (3) proteins involved in the regulation of cell structure and morphology (Table 3); (4) proteins involved in carbohydrate and energy metabolism (Table 4); and (5) proteins with other enzymatic activities (Table 5).

### 3.2. Proteins Responsible for Vesicular Trafficking and Endocytosis

**Figure 2:**
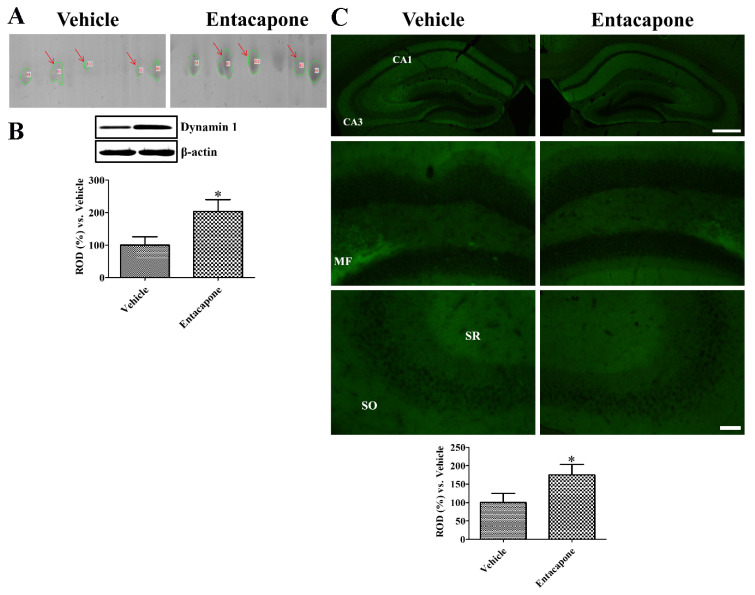
Dynamin 1 expression in the hippocampus of vehicle- and entacapone-treated groups based on proteomic (**A**), western blot (**B**), and immunohistochemical (**C**) study. Dynamin 1 are detected in three different spots using proteomic analysis and western blot analysis shows significant increases in dynamin 1 protein levels of entacapone-treated group compared to that in the control group. Western blot assays were performed in at least triplicate and bar graph represents the mean ± standard deviation. Dynamin 1 immunoreactivity is shown in the stratum radiatum (SR) and oriens (SO) of hippocampal CA1-CA3 region and mossy fibers (MFs). Scale bar = 400 μm (low magnification), 50 μm (high magnification). Three sections, 150 μm apart, are selected between 1.7 and 2.3 mm caudal to the bregma in each animal. Relative optical densities (ROD) are expressed as a percentage of the value of dynamin 1 protein levels and immunoreactivity in the vehicle-treated group (*n* = 5 in each group; * *p* < 0.05, significantly different from the vehicle-treated group). The bars indicate the mean values ± standard deviation.

**Figure 3:**
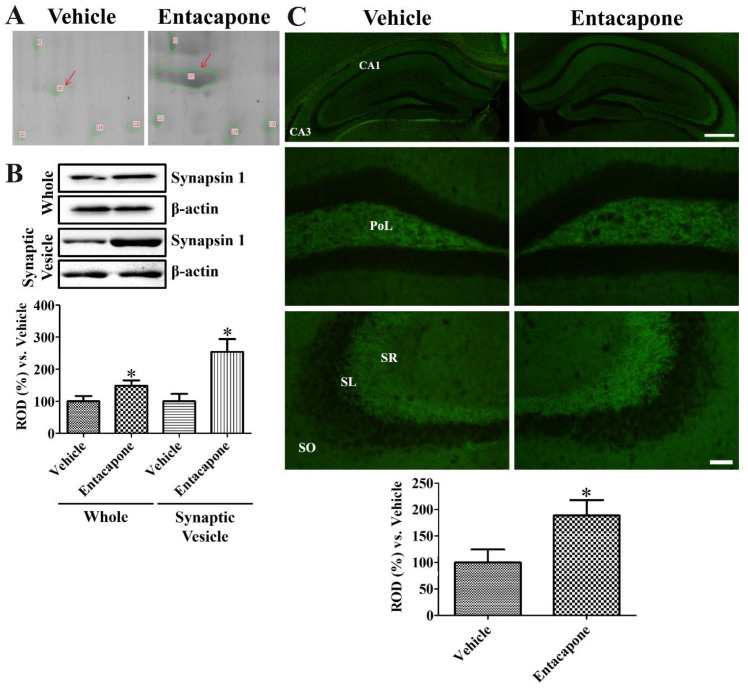
Synapsin I expression in the hippocampus of vehicle- and entacapone-treated groups based on proteomic (**A**), western blot (**B**), and immunohistochemical (**C**) study. Synapsin I spots were detected in proteomic gels and synapsin I protein levels are significantly increased in the hippocampus from whole and synaptic vesicle fractions. Western blot assays were performed in at least triplicate and bar graph represents the mean ± standard deviation. Synapsin I immunoreactivity is detected in the polymorphic layer (PoL) of dentate gyrus and stratum lucidum (SL) of CA3 region. Scale bar = 400 μm (low magnification), 50 μm (high magnification). Three sections, 150 μm apart, are selected between 1.7 and 2.3 mm caudal to the bregma in each animal. Relative optical densities (ROD) are expressed as a percentage of the value of synapsin I protein levels and immunoreactivity in the vehicle-treated group (*n* = 5 in each group; * *p* < 0.05, significantly different from the vehicle-treated group). The bars indicate the mean values ± standard deviation.

**Figure 4:**
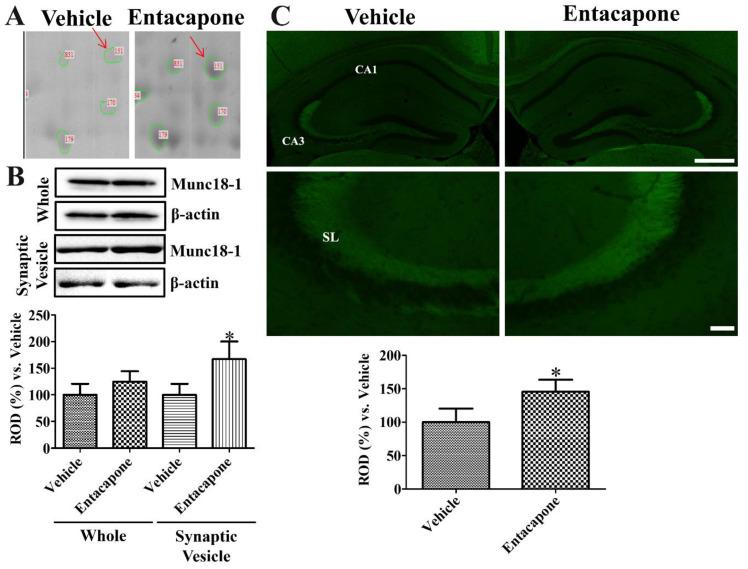
Munc18-1 expression in the hippocampus of vehicle- and entacapone-treated groups based on proteomic (**A**), western blot (**B**), and immunohistochemical (**C**) study. Munc18-1 was detected in a single spot. Munc18-1 protein levels were not significantly different between the hippocampi of vehicle- and entacapone-treated groups, while protein levels are significantly increased in the synaptic vesicle fraction of entacapone-treated group compared to that in the control group. Relative optical density in the entacapone-treated group versus that in the vehicle-treated group is shown in percentages for western blot analysis (* *p* < 0.05, significantly different from the vehicle-treated group). Western blot assays were performed in at least triplicate and bar graph represents the mean ± standard deviation. Munc18-1 immunoreactivity is observed in the stratum lucidum (SL) of CA3 region. Scale bar = 400 μm (low magnification), 50 μm (high magnification). Three sections, 150 μm apart, are selected between 1.7 and 2.3 mm caudal to the bregma in each animal. Relative optical densities (ROD) are expressed as a percentage of the value of Munc18-1 protein levels and immunoreactivity in the vehicle-treated group (*n* = 5 in each group; * *p* < 0.05, significantly different from the vehicle-treated group). The bars indicate the mean values ± standard deviation.

First, treatment with entacapone induced expressional changes in proteins that regulate vesicular trafficking and endocytosis. Dynamin 1 (+4.0-, +2.7-, and +25.9-fold change in spots no. 62, 65, and 810, respectively) was identified in three spots (Figure 2). Expression of synapsin I (+20.2-fold change in spot no. 105; −4.0-fold change in spot no. 106) (Figure 3) and synapsin II (+2.6-fold change) were significantly changed. The expression levels of the following proteins responsible for endocytosis, protein transport, vesicular trafficking, and neurotransmitter release also changed significantly: *N*-ethylmaleimide sensitive fusion protein (NSF, +2.1-fold), vesicle-fusing ATPase (+3.1-fold), Munc18-1 (+4-fold) (Figure 4), Eps15 homology (EH) domain containing protein 3 (+2.3-fold), Rab GDP dissociation inhibitor beta (+2.2-fold), adaptin ear-binding coat-associated protein 1 (+2.5-fold), and synaptosomal associated protein 25 (SNAP-25) (+2.5-fold) (Table 1).

### 3.3. Proteins Responsible for Cellular Signaling and Processes

We identified proteins that are implicated in cellular signaling and diverse cellular processes, such as the cell cycle, mitosis, differentiation, and transformation. The expression levels of the following proteins were changed: transformation/transcription domain-associated protein (+3.9-fold), nuclear receptor-binding SET-domain protein 1 (+4.7-fold), COP9 signalosome complex subunit 4 (+2.6-fold), guanine nucleotide-binding protein alpha 11 subunit (+2.8-fold), fizzy/cell division cycle 20 related 1 (+2.0-fold), valosin-containing protein (−2.9-fold), protein tyrosine phosphatase (−2.2-fold), and tyrosine 3-monooxygenase/tryptophan 5-monooxygenase activation protein (−2.1-fold) (Table 2).

### 3.4. Proteins Responsible for the Regulation of Cell Structure and Morphology

Proteins that regulate the morphology and cytoskeletal organization, and thus affect neuritogenesis and cell migration, were identified. The expression levels of the following proteins changed significantly: titin isoform N2-B (+2.1-fold), dihydropyrimidinase-related protein 1 and 2 (+2.1- and −3.7-fold in spots no. 179 and 152, respectively), tubulin alpha-1C chain (−2.9-fold), tubulin beta-4A chain (+4.7-fold), tetratricopeptide repeat protein 23 isoform 2 (+2.0-fold), septin 2 (+2.0-fold), actin monomer-binding protein twinfilin-1 (+2.3-fold), formin-like protein 1 (+3.0-fold), actin-related protein 2/3 complex subunit 2 (+2.0-fold), axonemal dynein heavy chain 5 (+2.0-fold), myosin-7 (+2.4-fold), spectrin alpha chain (−2.1-fold), and neurofilament light polypeptide (−5.8-fold) (Table 3).

### 3.5. Proteins Regulating Energy Metabolism

Proteins implicated in carbohydrate and energy metabolism were identified from spots. The expression levels of the following metabolic enzymes were altered: mitochondrial aconitase 2 (+5.1-fold), fumarate hydratase 1 (+2.3-fold), alpha-enolase (+2.2- and +2.1-fold in spots no. 318 and 323, respectively), mitochondrial precursor of 3-hydroxyisobutyryl-CoA hydrolase (+2.7-fold), 3′(2′),5′-bisphosphate nucleotidase 1 (+3.3-fold), UMP-CMP kinase (+2.3-fold), muscle isoform M1 of pyruvate kinase (+2.6-fold), transketolase (+2.4-fold), alpha subunit of the H^+^ transporting ATP synthase of the mitochondrial F1 complex (−2.4-fold), dihydrolipoamide S-acetyltransferase precursor (−2.2-fold), calcium-binding protein 39 (+2.7-fold), and pyrophosphatase 2 (−2.1-fold) (Table 4).

### 3.6. Proteins with Various Enzymatic Activities

Proteins with various enzymatic activities were identified from spots. Expression levels of the following enzymes changed: isoaspartyl peptidase/L-asparaginase (+3.6-fold), mGSTA4-4 (+2.6-fold), leukotriene-A4 hydrolase (+2.0-fold), Ccdc80 protein (+2.3-fold), glutamate dehydrogenase 1 (+4.0-fold), protein phosphatase methylesterase 1 (+3.2-fold), cytoplasmic aspartate aminotransferase (+2.0-fold), monoglyceride lipase (+2.2-fold), pyridoxal phosphate phosphatase (PLPP, +2.2-fold), and carbonyl reductase [NADPH] 3 (+2.0-fold) (Table 5).

### 3.7. Validation of Protein Level Changes for Dynamin 1, Synapsin I, and Munc18-1

To validate the proteins and to visualize the locations of proteins identified with 2-DE and MALDI-TOF MS, we performed western blot analysis and immunohistochemical staining, respectively, for dynamin 1, synapsin I, and Munc18-1 (Figure 2). Dynamin was detected in three different spots in 2-DE (Figure 2A). Western blot analysis showed that dynamin 1 levels in the entacapone-treated group significantly increased to 203.5% of vehicle-treated group (Figure 2B). Dynamin 1 immunoreactivity in vehicle- and entacapone-treated groups was found in the stratum oriens and radiatum of the hippocampal CA1-3 region and outer molecular layer of the dentate gyrus. Dynamin immunoreactivity in the entacapone-treated group was significantly increased to 175.2% of vehicle-treated group (Figure 2C).

Based on 2-DE, synapsin I spot was larger in entacapone-treated group than in the vehicle-treated group (Figure 3A). Western blot analysis demonstrated that synapsin I protein levels were significantly increased to 148.8% and 254.0% of the vehicle-treated group in the whole hippocampus and synaptic vesicle fractions, respectively (Figure 3B). Synapsin I immunoreactivity in vehicle- and entacapone-treated groups was observed in the stratum lucidum of the hippocampal CA3 regions and mossy fibers in the dentate gyrus. Synapsin I immunoreactivity in the entacapone-treated group was significantly increased to 188.8% of vehicle-treated group (Figure 3C).

Dense Munc18-1 spot was detected in the 2-DE gels of entacapone-treated group compared to that in the vehicle-treated group (Figure 4A). Munc18-1 protein levels based on western blot study did not show any significant differences in the hippocampal homogenates of vehicle- and entacapone-treated groups. However, in the synaptic vesicle fraction, Munc18-1 showed significantly higher levels (167.1% of vehicle-treated group) in the entacapone-treated group (Figure 4B). Munc18-1 immunoreactivity in vehicle- and entacapone-treated groups was detected in the stratum lucidum of the hippocampal CA3 regions. Munc18-1 immunoreactivity in the entacapone-treated group was significantly increased to 145.7% of vehicle-treated group (Figure 4C).

## 4. Discussion

In the present study, we performed 2-DE and MALDI-TOF MS to elucidate the effects of entacapone on protein expression in the mouse hippocampus. We determined the identity of 76 protein spots, and the identified proteins were categorized into five groups.

### 4.1. Vesicular Trafficking and Endocytosis

Dynamin 1 was identified from three spots, and its expression level was markedly increased after entacapone administration. Dynamin 1, highly enriched in presynaptic terminals, is a GTP-binding protein, and is implicated in clathrin-mediated endocytosis [24]. In addition, the EH domain-containing protein 3 and adaptin ear-binding coat-associated protein 1 regulate endosomal transport and endocytosis [25,26]. Expressional changes in proteins belonging to this category support that COMT inhibition may influence the release of neurotransmitters in hippocampal neurons as well as the expression and elimination of synaptic molecules in the synaptic cleft.

### 4.2. Cellular Signaling and Various Cellular Processes Including the Cell Cycle, Mitosis, and Differentiation

Proteins categorized into this group are involved in various cellular processes, including signal transduction and regulation of the cell cycle. Transformation/transcription domain-associated protein is an adaptor protein with histone acetyltransferase activity and controls cell cycle progression in adult neurogenesis [27]. Additionally, it is implicated in the transcription of Myc, which plays important roles in the regulation of cell cycle, signaling, and cell growth [28]. Similarly, nuclear receptor-binding SET-domain protein 1 and guanine nucleotide-binding protein are implicated in cellular signaling [29,30]. In addition, COP9 signalosome complex subunit 4 and fizzy/cell division cycle 20 related 1 are key players in the control of the cell cycle, and they perform additional cellular functions [31,32]. Protein tyrosine phosphatase eliminates the phosphate groups from tyrosine residues of proteins, and acts as a key regulator of various cellular processes, such as the cell cycle, signal transduction, proliferation, and differentiation [33,34]. Tyrosine 3-monooxygenase/tryptophan 5-monooxygenase activation protein (14-3-3) is implicated in the mitogen-activated protein kinase pathway and regulates cell division and apoptosis [35]. In addition, the inhibition of 14-3-3 promotes neuronal loss and synaptic deficits in brain diseases [36,37]. Double knockout of 14-3-3ε and 14-3-3ζ, not its single knockout, significantly increases the proliferating cells during embryogenesis in mice [35]. Expressional changes in these proteins support the idea that entacapone treatment promotes the cell proliferation and neuroblast differentiation with consistent to our previous study that entacapone enriched the hippocampal functions and neurogenesis in mice [20].

### 4.3. Cell Structure and Morphology

Administration of entacapone induced expressional changes in proteins that are responsible for the construction of cell structure and the induction of cell migration. The expression of tubulin alpha and beta chains, which are major constituents of microtubules and the cytoskeleton, was changed, and the actin-associated proteins twinfilin 1 and actin-related protein 2/3 complex subunit 2 had increased expression levels. It has been reported that twinfilin 1 plays an essential role in the regulation of cytoskeletal structure [38], and actin-related protein 2/3 complex subunit 2 is a regulator of cell migration in neural stem cell-derived oligodendrocytes in electric fields [39]. Depletion of *twinfilin* gene in flies showed defects in axonal growth of brain [40,41]. Expression of the dihydropyrimidinase-related proteins 1 and 2 was changed; these proteins are necessary for cytoskeleton remodeling, and they play a role in neuroprotection and synaptic plasticity [42,43]. In addition, the expression of spectrin, an actin crosslinking scaffold protein, and the expression of neurofilament light polypeptide was significantly changed [44]. Expressional changes in proteins belonging to this category support that COMT inhibition modulates protein related to cell structure and migration in the hippocampus, which promotes the hippocampal neurogenesis described in the previous study [20].

### 4.4. Energy Metabolism

After the administration of entacapone, the expression of proteins associated with carbohydrate and energy metabolism was significantly changed. Both alpha enolase, which was identified from two spots showing an increase upon entacapone treatment, as well as pyruvate kinase are glycolytic enzymes, and dihydrolipoamide S-acetyltransferase precursor participates in the metabolic pathway that links glycolysis and the tricarboxylic acid (TCA) cycle. Altered expression of proteins that regulate energy production might indicate that cellular functions with a high energy demand were initiated after entacapone treatment. In our previous study, we demonstrated that treatment with entacapone increased cell proliferation and neuroblast differentiation in the mouse hippocampus [20]. It has been reported that glucose metabolism and neurogenesis are closely related [45,46], and energy production is necessary not only for synaptic transmission but also for the proliferation, survival, and differentiation of neural stem cells [45]. We previously demonstrated that the neuronal type of glucose transporter plays an important role in postnatal development and neurogenesis in the hippocampus [47]. In addition, the treatment with phosphoglycerate mutase 1, a glycolytic enzyme, significantly increases the proliferation cells and neuroblasts in the hippocampus by promoting the phosphorylation of cAMP response element-binding protein [48]. A recent study showed entacapone directly bound to fat mass and obesity-associated gene (FTO) and inhibited the activity of FTO [49]. In addition, entacapone reduced the body weight and blood glucose concentration in obese mice [49]. In the present study, altered expression of proteins implicated in energy metabolism may increase the hippocampal neurogenesis as described in the previous study [20].

### 4.5. Various Enzymatic Activities

The proteins categorized into this group mediate various enzymatic reactions. In this category, proteins essential for glutamate synthesis were also identified. Glutamate is an excitatory neurotransmitter that plays an important role in the neuronal activation of the hippocampus [50,51].

Activation of dopaminergic neurotransmission induces long-term synaptic plasticity by facilitating glutamatergic NMDA receptors [52], and inhibition of dopamine-receptor signaling impaired passive avoidance memory and induction of long-term potentiation (LTP) in the hippocampus [53]. Glutamate receptors are closely involved in LTP-induced synaptic enhancement [54] and dopamine signal is required in the late LTP [55]. Glutamate dehydrogenase 1 plays a key role in the synthesis and degradation of glutamate, and is involved in hippocampal synapse formation [56,57]. Aspartate aminotransferase (AST) is closely involved in catalyzing glutamate formation and oxidation in brain [58], and administration of cytosolic AST increased axonal growth in cultured cortical neurons and enhanced memory function [59]. In the present study, the inhibition of COMT changes the metabolism of glutamate and this may be related to promotion of synaptic formation and axonal growth.

### 4.6. Validation of Synaptic Vesicle Trafficking-Related Proteins

In this study, we confirmed that dynamin 1 immunoreactivity was found in the presynaptic terminals of the hippocampus, and entacapone treatment significantly increased dynamin 1 expression in the hippocampus, which controls vesicular trafficking and endocytosis. Aged rodents show significantly lower levels of dynamin 1 in the hippocampus compared to adult rodents [60,61]. In addition, dynamin 1 expression is significantly decreased in an animal model of Alzheimer’s disease [62,63].

Munc18-1 is a member of the synaptic vesicle fusion protein complex, and has an important role in the exocytosis of neurotransmitters [64] and neurite outgrowth during development [65]. Munc18-1 interacts with the SNARE complex [66,67] and facilitates the zippering of *trans*-SNARE complexes and promotes the SNARE-dependent membrane fusion in synaptosomes [68]. In contrast, knockdown of Munc18-1 shows abnormalities in cortical development [69] and diminished secretion of brain-derived neurotrophic factor [70]. In the present study, we observed Munc18-1 expression in the stratum lucidum in the hippocampus and its significant increase by entacapone treatment.

Synapsin I, a presynaptic marker protein, acts as a modulator of neurotransmitter release and synaptic vesicles [71], and inhibition of synapsin I decreased activity of both excitatory and inhibitory synapses in hippocampal cells [72]. Knockout of all synapsins showed lower serotonin levels in the hippocampus and behavioral dysfunctions [73]. Neurodegenerative diseases involve synaptic dysfunction, and synapsin I was significantly decreased in plasma neuronal-derived exosomes of Alzheimer’s disease patients [74]. In the present study, we observed synapsin I immunoreactivity in the stratum lucidum of the hippocampal CA3 regions and mossy fibers in the dentate gyrus, and synapsin I expression was significantly elevated after entacapone treatment.

Neurotransmission is modulated by various factors such as presynaptic vesicular exocytosis, postsynaptic receptor expression, recycling of neurotransmitters, and activation of glial cells [75,76]. Administration of a COMT inhibitor activates dopaminergic neurotransmission in cerebral cortex [77]. In the hippocampus, dopaminergic innervation is well-detected and modulates hippocampal network and specific types of dopaminergic receptors are expressed in each hippocampal subregion [78,79]. In the present study, we confirmed expressional changes of the proteins which are closely related to vesicular trafficking in the hippocampal subregions following entacapone treatment. We are not sure that the proteins, which showed increased expression level in CA1, CA3, and dentate gyrus in this study, are expressed by hippocampal glutamatergic neurons, but increased level of vesicle trafficking-related proteins in the hippocampus is closely involved in synaptic plasticity and behavioral functions [80].

In conclusion, proteomic analysis indicated that entacapone induced changes in the expression of proteins involved in synaptic transmission, cellular processes, cellular signaling, the regulation of cytoskeletal structure, energy metabolism, and various other cellular reactions. In particular, increased vesicle trafficking-related proteins may be closely related to enhance synaptic plasticity and hippocampal functions.

## Figures and Tables

**Table 1:** Differentially expressed proteins related to vesicular trafficking after entacapone treatment in the hippocampus.

Spot no.	Accession no.	Protein Name	Score	Nominal Mass (Da)	pI Value	Matched Peptide	Sequence Coverage (%)	Average Ratio
**62**	gi|148676592	Dynamin 1	73	97,035	7.6	20	23	4.0
**65**	gi|32172431	Dynamin 1	195	98,140	7.61	39	42	2.7
**810**	gi|148676592	Dynamin 1	225	97,035	7.6	40	43	25.9
**105**	gi|148668411	Synapsin I	137	69,798	9.84	22	44	20.2
**118**	gi|55154394	Synapsin II	67	63,487	8.59	12	29	2.6
**107**	gi|148702256	*N*-Ethylmaleimide sensitive fusion protein	109	78,329	6.68	25	35	2.1
**108**	gi|146345470	Vesicle-fusing ATPase	71	83,131	6.52	18	28	3.1
**151**	gi|3810884	Munc18-1	77	67,906	6.41	22	38	4.0
**187**	gi|172044688	Eps15 homology (EH) domain containing protein 3	109	60,840	6.03	24	50	2.3
**310**	gi|13626886	Rab GDP dissociation inhibitor beta	146	51,018	5.93	26	51	2.2
**423**	gi|62287163	Adaptin ear-binding coat-associated protein 1	83	29,678	5.97	11	47	2.5
**594**	gi|46397725	Synaptosomal-associated protein 25	68	23,528	4.66	13	65	2.5
**106**	gi|73920802	Synapsin I	155	74,223	9.81	24	46	−4.0

**Table 2:** Differentially expressed proteins related to cell signaling and processes after entacapone treatment in the hippocampus.

Spot no.	Accession no.	Protein Name	Score	Nominal Mass (Da)	pI Value	Matched Peptide	Sequence Coverage (%)	Average Ratio
**277**	gi|124486949	Transformation/transcription domain-associated protein	70	439,911	8.51	37	12	3.9
**324**	gi|148709230	Nuclear receptor-binding SET-domain protein 1	71	267,700	8.94	30	16	4.7
**381**	gi|55976221	COP9 signalosome complex subunit 4	113	46,541	5.57	24	55	2.6
**399**	gi|15029890	Guanine nucleotide binding protein, alpha 11	79	42,189	5.7	15	48	2.8
**422**	gi|148699469	Fizzy/cell division cycle 20 related 1	67	46,888	9.74	12	27	2.0
**803**	gi|148670554	Valosin containing protein	103	91,675	5.26	26	30	−2.9
**48**	gi|148698569	Protein tyrosine phosphatase	68	147,039	6.52	19	20	−2.2
**563**	gi|148676868	Tyrosine 3-monooxygenase/tryptophan 5-monooxygenase activation protein, zeta polypeptide	92	29,240	4.71	16	42	−2.1

**Table 3:** Differentially expressed proteins related to cell structure and morphology after entacapone treatment in the hippocampus.

Spot No.	Accession no.	Protein Name	Score	Nominal Mass (Da)	pI Value	Matched Peptide	Sequence Coverage (%)	Average Ratio
**92**	gi|77812699	Titin isoform N2-B	76	3,004,899	6.24	85	4	2.1
**179**	gi|3122030	Dihydropyrimidinase-related protein 1	124	62,471	6.63	21	51	2.2
**237**	gi|146345529	Tubulin beta-4A chain	119	50,010	4.78	22	46	4.7
**242**	gi|270309132	Tetratricopeptide repeat protein 23 isoform 2	67	50,391	8.36	13	34	2.0
**849**	gi|9957546	Septin 2	94	49,050	5.74	18	39	2.0
**393**	gi|30421118	Actin monomer-binding protein twinfilin 1	67	40,368	6.33	10	38	2.3
**451**	gi|67460975	Formin-like protein 1	69	122,724	5.62	20	21	3.0
**514**	gi|110825706	Actin-related protein 2/3 complex subunit 2	74	34,450	6.84	14	47	2.0
**567**	gi|18449111	Axonemal dynein heavy chain 5	68	531,101	5.81	50	15	2.0
**850**	gi|81871557	Myosin 7	70	223,539	5.59	27	20	2.4
**28**	gi|295054271	Spectrin alpha chain	87	283,519	5.2	36	18	−2.1
**125**	gi|116283387	Neurofilament light polypeptide	140	57,848	4.78	24	40	−5.8
**152**	gi|94730376	Dihydropyrimidinase-related protein 2 (CRMP2)	78	62,638	5.95	14	26	−3.7
**848**	gi|55977482	Tubulin alpha-1C chain	76	50,562	4.96	16	48	−2.9

**Table 4:** Differentially expressed proteins related to energy metabolism after entacapone treatment in the hippocampus.

Spot No.	Accession no.	Protein Name	Score	Nominal Mass (Da)	pI Value	Matched Peptide	Sequence Coverage (%)	Average Ratio
**82**	gi|63101587	Aconitase 2, mitochondrial	184	86,185	8.08	30	41	5.1
**303**	gi|13543801	Fumarate hydratase 1	110	54,564	9.12	24	50	2.3
**318**	gi|13637776	Alpha-enolase	98	47,453	6.37	20	50	2.2
**323**	gi|13637776	Alpha-enolase	79	47,453	6.37	15	38	2.1
**364**	gi|22122625	3-Hydroxyisobutyryl-CoA hydrolase, mitochondrial precursor	68	43,295	8.16	13	35	2.7
**408**	gi|46396056	3′(2′),5′-Bisphosphate nucleotidase 1	70	33,517	5.54	12	43	3.3
**653**	gi|23821758	UMP-CMP kinase	74	22,379	5.68	12	61	2.3
**835**	gi|359807367	Pyruvate kinase, muscle isoform M1	75	58,461	6.69	21	40	2.6
**837**	gi|148692809	Transketolase	75	56,273	7.15	15	42	2.4
**899**	gi|148708308	Calcium binding protein 39	68	40,831	6.23	15	35	2.7
**824**	gi|16580128	Dihydrolipoamide S-acetyltransferase precursor	69	59,389	5.71	20	31	−2.2
**906**	gi|148680234	Pyrophosphatase (inorganic) 2	68	35,754	8.8	12	47	−2.1

**Table 5:** Differentially expressed proteins related to enzyme activity after entacapone treatment in the hippocampus.

Spot No.	Accession no.	Protein Name	Score	Nominal Mass (Da)	pI Value	Matched Peptide	Sequence Coverage (%)	Average Ratio
**119**	gi|81875980	Isoaspartyl peptidase/L-asparaginase	69	32,385	7.56	12	37	3.6
**632**	gi|6137391	mGSTA4-4	74	25,428	7	14	60	2.6
**827**	gi|341941138	Leukotriene-A4 hydrolase	105	69,634	5.98	22	39	2.0
**829**	gi|118600953	Ccdc80 protein	71	57,797	10.26	14	40	2.3
**858**	gi|148692928	Glutamate dehydrogenase 1	144	54,527	7.66	24	44	4.0
**875**	gi|108935875	Protein phosphatase methylesterase 1	69	42,628	5.67	15	38	3.2
**887**	gi|338817898	Aspartate aminotransferase, cytoplasmic	165	46,504	6.68	21	60	2.0
**911**	gi|148666837	Monoglyceride lipase	82	37,330	7.77	19	45	2.2
**921**	gi|44888293	Pyridoxal phosphate phosphatase	70	31,891	5.53	13	48	2.2
**926**	gi|81914662	Carbonyl reductase [NADPH] 3	67	31,333	6.15	11	45	2.0
